# Lyophilized horizontal platelet rich fibrin promotes the healing of infected burns/wounds by modulating macrophage polarization and fibroblast migration

**DOI:** 10.3389/fbioe.2025.1666265

**Published:** 2025-09-29

**Authors:** Jihua Chai, Detian Miao, Richard J. Miron, Nima Farshidfar, Shuo Xu, Xiaoxin Zhang, Yulan Wang, Yi Bai, Hualing Sun

**Affiliations:** ^1^ State Key Laboratory of Oral and Maxillofacial Reconstruction and Regeneration, Key Laboratory of Oral Biomedicine Ministry of Education, Hubei Key Laboratory of Stomatology, School and Hospital of Stomatology, Taikang Center for Life and Medical Sciences, Wuhan University, Wuhan, China; ^2^ Department of Stomatology, Binzhou Medical University Hospital, Binzhou, Shandong, China; ^3^ Department of Periodontology, University of Bern, Bern, Switzerland

**Keywords:** lyophilized, horizontal centrifugation, platelet rich fibrin, infected burned wound, macrophage

## Abstract

**Background:**

The treatment of infected burns is a major clinical challenge. Platelet-rich fibrin produced via horizontal centrifugation (H-PRF) has been characterized with antimicrobial and tissue regenerative properties. Noteworthy, its lyophilized form (Ly-H-PRF), which can be conveniently preserved, may also have similar regenerative potential for the treatment of infected burns. The aim of this study was to investigate whether Ly-H-PRF could promote wound healing and its regulatory mechanism in various *in vitro* and *in vivo* models simulating infected burns/wounds.

**Methods:**

Venous blood of healthy volunteers was drawn, horizontally centrifuged at 700 RCF for 8 min, and lyophilized to obtain Ly-H-PRF. Ly-H-PRF was dissolved in culture medium, and its antimicrobial effects were evaluated on *Staphylococcus aureus* (S.a) and *Escherichia coli* (E.c) by the poured-plate method. Furthermore, the effects of Ly-H-PRF on the cell cycle and polarization of macrophages after lipopolysaccharide (LPS) stimulation were also investigated by fluorescence staining and flow cytometry. The effects of Ly-H-PRF on skin fibroblasts after LPS culture were also tested by flow cytometry, a transwell assay, and a scratch assay. Lastly, a mouse second-degree burn model was used with four groups, including 1) PBS, 2) S.a infection, 3) S.a infection + burn ointment, and 4) S.a infection + Ly-H-PRF. Histological assessment was used to investigate the healing of the burn wound tissues, inflammatory cell infiltration, neo-collagenous tissues, and macrophage polarization after 5 days.

**Results:**

Ly-H-PRF effectively inhibited the growth of S.a and E.c. It also protected macrophages from LPS-stimulated apoptosis and reduced LPS-induced macrophage M1 polarization and promoted M2 polarization. Ly-H-PRF further protected fibroblasts from LPS-stimulated apoptosis and facilitated fibroblasts migration. In the *in vivo* burn wound model, S.a infections led to a greater wound enlargement and ulceration at 5 days post-op, and routine use of burn ointment was less effective than treatment of infected wounds with Ly-H-PRF. Noteworthy, the Ly-H-PRF promoted wound healing, reduced inflammatory cell infiltration, and increased collagen synthesis.

**Conclusion:**

The present study demonstrated that Ly-H-PRF promoted the healing of infected burn wounds by exerting an antibacterial effect, regulating macrophage polarization, and promoting skin fibroblast migration. Our results provide a pre-theoretical basis for the clinical application of Ly-H-PRF as an economical and convenient treatment for infection control and to promote tissue healing in infected burn wounds.

## 1 Introduction

Burns are caused by various factors, including flames caused by fire, hot liquids, metals, chemicals, electric shocks, and radiation (Jeschke et al., 2020). They can lead to mortality or disability, dramatically reducing the patient’s wellbeing and quality of life (Behera et al., 2025). According to a report by the World Health Organization, approximately 300,000 people die from burns worldwide each year (Peck, 2011; G et al., 2022). Burns compromise the skin barrier, facilitating bacterial invasion. *Staphylococcus aureus* (S.a) is a common skin bacterium that can quickly colonize burn wounds and potentially develop into an infection (Hernandez et al., 2021). S.a is also one of the primary pathogens in hospital-acquired infections. In burn units, the risk of infection has been shown to increase during prolonged hospital stays, and when antibiotics are over-prescribed (Liu et al., 2022). If burn wounds become infected, immune dysregulation will subsequently occur, which has been shown to roughly double the mortality rate among patients (Lachiewicz et al., 2017). The key to managing burn wounds is to achieve complete repair and regeneration as quickly as possible, while minimizing infection, contracture, and scar formation (Wang et al., 2018). Common clinical treatments such as wound dressing care, negative pressure wound therapy, debridement, and skin grafting have often been applied to accelerate wound healing, though they can also lead to sequelae such as scar hyperplasia and recurrent ulceration (Shu et al., 2021; Wang et al., 2018).

In recent years, clinical researchers have increasingly recognized the critical importance of growth factor delivery in promoting wound healing in burn areas (Legrand and Martino, 2022). Platelet concentrates derived from the patient’s own blood represent a more cost-effective option compared to using recombinant growth factors. When applied to wounds, it releases growth factors such as platelet-derived growth factor (PDGF), transforming growth factor beta (TGF-β), and vascular endothelial growth factor (VEGF), promoting vascularization and tissue regeneration (Miron et al., 2025; Farshidfar et al., 2025a). Currently, platelet-rich fibrin (PRF), a second-generation platelet concentrate, has gained widespread attention for its significant benefits in the treatment of burns and various acute/chronic wounds (Bai et al., 2023; Miron et al., 2017; Farshidfar et al., 2025b; Pinto et al., 2000; Bilgen et al., 2021). Results have demonstrated that PRF can improve infected wounds in an animal model (Silveira et al., 2023). PRF has also exhibited strong anti-inflammatory properties, which can help modulate inflammatory response and further support tissue regeneration and healing (Strauss et al., 2020; Kargarpour et al., 2021; Kargarpour et al., 2022). Additionally, PRF possesses promising antimicrobial activity, which could potentially reduce the risk of infection (Moraschini et al., 2000). Research has further demonstrated that platelet extracts alone are even capable of accelerating tissue formation, enhancing wound healing, and improving burn wound closure, thereby shortening the recovery time and reducing the potential for scarring caused by burns (Farshidfar et al., 2025b; Pinto et al., 2000).

In recent years, it was shown that a novel preparation of PRF using horizontal centrifugation (H-PRF) leads to a more favorable way to optimize platelet counts and growth factor accumulation (Miron et al., 2019; Miron et al., 2000; Qiu et al., 2023; Quirynen et al., 2000; Farshidfar et al., 2000). This method, by employing horizontal rather than fixed-angle centrifugation, yields a more uniform distribution of viable cells with enhanced activity (Miron et al., 2000; Farshidfar et al., 2000; Fujioka-Kobayashi et al., 2021). Additionally, H-PRF has demonstrated a significantly stronger antibacterial effect compared to fixed-angle PRF (Feng et al., 2020), and the literature has also confirmed its anti-inflammatory properties (Nasirzade et al., 2020).On the other hand, while the use of PRF membranes has shown efficacy in treating burns, it requires an immediate blood draw, which may not always be possible or so readily available in fresh burn victims. Additionally, it can be challenging to obtain the necessary volume of blood in a single session for larger wounds. Lyophilized PRF (Ly-PRF), prepared through freeze-drying, can be stored at various temperatures for extended periods, both with and even without refrigeration. It is conveniently rehydrated with saline or another isotonic solution when needed, making it highly practical. Freeze-drying technology allows PRF to have an extended service life that is no longer limited to the short-term use requirements of fresh PRF, thereby increasing its flexibility of use (Zhang et al., 2017; Liu et al., 2019).

**SCHEME 1:**
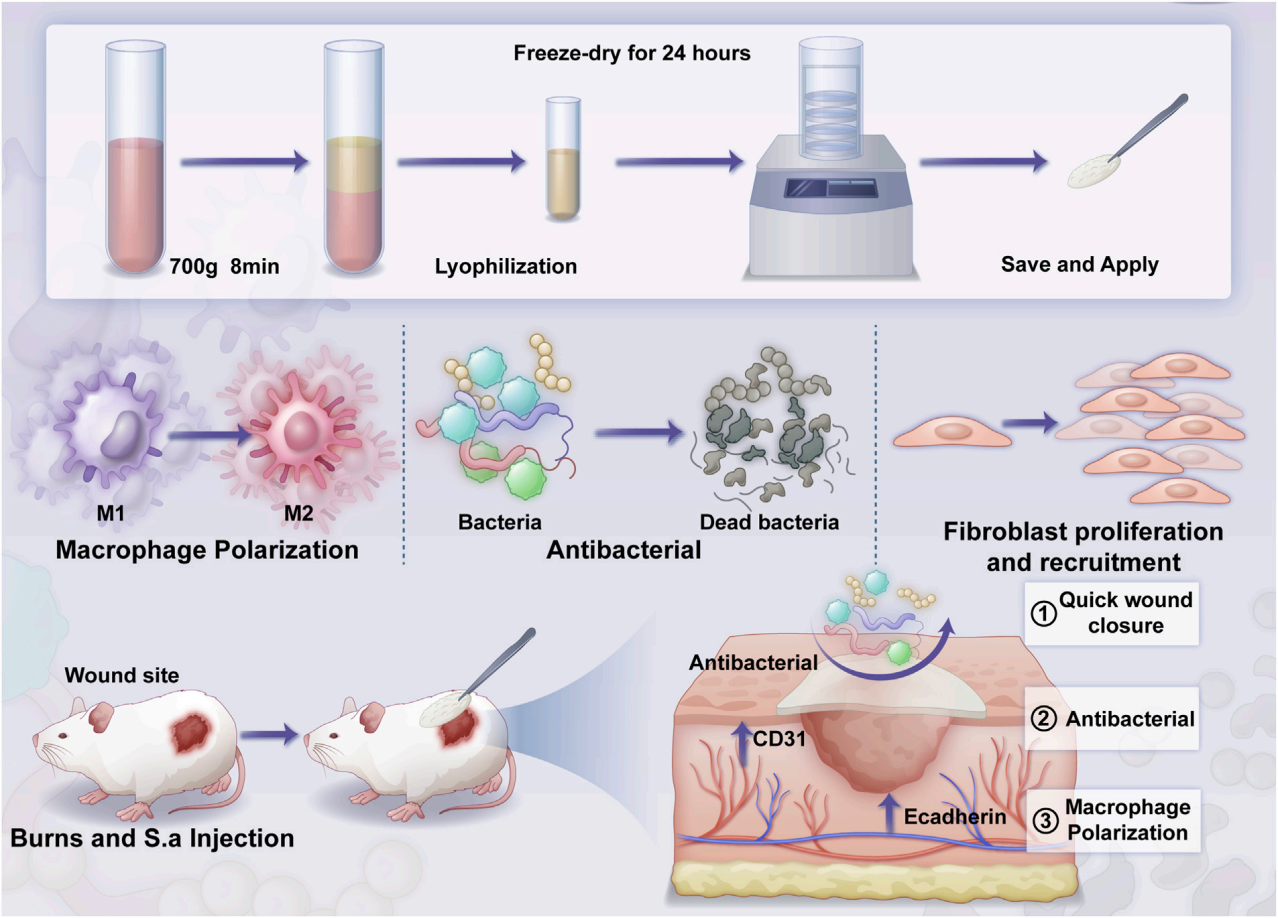
The overall structure of the article includes: material preparation, *in vitro* validation, and *in vivo* validation.

Therefore, the aim of this study was to explore the therapeutic efficacy of a lyophilized H-PRF (Ly-H-PRF) in a co-infected burn model, mainly by exploring its immunomodulatory effecton macrophages through *in vitro* assays, and its repairing effecton scald wounds through murine infected burn wound models (Scheme 1).

## 2 Materials and methods

### 2.1 Preparation of ly-H-PRF

This research was carried out with the explicit consent obtained from the School and Hospital of Stomatology, Wuhan University (MRI2023-LACA14, WDKQ 2024A28). Peripheral blood was obtained from six healthy volunteers (aged 25–40 years) in glass blood collection tubes (Plasmatrident, Weiyin Technology Co., Ltd., Wuhan, China). The collected peripheral blood was subjected to centrifugation in a horizontal centrifuge with the centrifugation parameters of 700 g for 8 min, and the upper plasma layer was collected, and the volume was recorded after centrifugation. Then the liquid H-PRF was rapidly frozen and thawed 5 times in liquid nitrogen and in a 37 °C water bath, and then freeze-dried after centrifugation to remove cellular residues. Then, the Ly-H-PRF was obtained in its solid state as previously described (Qian et al., 2022). 5% of the Ly-H-PRF was dissolved in the same volume as the original volume and used for the experiments before the *in vitro* experiments, then photos were taken (Ngah et al., 2021). Scanning electron microscopy (SEM; Tescan MIRA, Czech Republic) was used to observe Ly-H-PRF at different magnifications.

### 2.2 *In vitro* antibacterial activity

The antimicrobial activity of Ly-H-PRF was determined by the poured-plate method as previously described (Terrones-Fernandez et al., 2023). 20 μL Ly-H-PRF solution was incubated with 180 μL S.a or *Escherichia coli* (E.c) suspensions (10^7^ CFU/mL) for 24 h, then the absorbance at OD = 600 nm at 0 and 24 h was recorded. After incubation, a 20 μL mixture of Ly-H-PRF and bacterial solution was diluted 100 times using BHI broth and then spreaded onto BHI agar plates. After culturing for 24 h at 37 °C, the bacterial colonies were photographed and counted. The remaining bacterial suspension was stained with STYO9/PI Live and Dead Bacteria Stain Kit (Bingene, China) and analyzed by fluorescence microscopy.

### 2.3 Cell culture

RAW 264.7 macrophage cells and L929 fibroblasts were cultured in high-glucose DMEM medium (HyClone, United States) supplemented with 10% fetal bovine serum (FBS) (Sigma, United States) and 1% penicillin/streptomycin (HyClone, United States). The cells were cultured at 37 °C in a humidified atmosphere containing 5% CO_2_. Upon reaching 80%–90% confluence, the cells were passaged by washing with 0.25% trypsin-EDTA solution.

### 2.4 Live/dead assay of macrophages

RAW 264.7 Cells were seeded in a 12-well plate with 15 mm diameter coverslips (NEST, United States) at a density of 1 × 10^5^ cells per well. The cells were treated with LPS (InvivoGen, 6 μg/mL) (Gibson and Walters, 2020) with or without 5% Ly-H-PRF for 24 h. After treatment, the cells were washed three times with PBS and stained with Calcein/PI cell viability and cytotoxicity assay kit (Beyotime C2015S-1, China). The staining was performed by incubating the cells at 37 °C for 30 min, followed by three washes with PBS. Coverslips were then mounted, and images were captured using confocal microscopy (Zeiss LSM880, Germany).

### 2.5 Flow cytometry analysis of macrophages and fibroblasts

RAW 264.7 Cells and L929 cells were seeded in a 12-well plate and treated with LPS (InvivoGen, 6 μg/mL) with or without 5% Ly-H-PRF for 24 h. Then the cells were trypsinized and washed three times with PBS before being centrifuged at 4,000 rpm for 5 min. The cells were then stained using the Calcein/PI cell viability and cytotoxicity assay kit (Beyotime) and incubated at 37 °C for 30 min. After incubation, cells were washed three times with PBS and resuspended in PBS for flow cytometric analysis using a BD Fortessa™ X-20 instrument (LSRFortessaX-20, BD). Data were analyzed using FlowJo 10.8.1.

For cell cycle analysis, RAW 264.7 cells were seeded in a 12-well plate at a density of 1 × 10^5^ cells per well and treated with LPS (InvivoGen, 6 μg/mL) with or without 5% Ly-H-PRF for 24 h. Subsequently, the cells were incubated in medium containing Hoechst 33342 (Thermo Fisher) for 90 min. After incubation, cells were washed three times with PBS, resuspended in PBS, and subjected to flow cytometric analysis.

For the apoptosis assay, RAW 264.7 cells and L929 cells were seeded in a 12-well plate and treated with LPS (InvivoGen, 6 μg/mL) with or without 5% Ly-H-PRF for 24 h Stained using the APC-Annexin V/PI apoptosis kit (Elabscience, China) and incubated at 4 °C for 30 min. After staining, cells were washed three times with PBS, resuspended in PBS, and subjected to flow cytometric analysis.

### 2.6 Macrophage polarization assay

RAW 264.7 cells were seeded in a 24-well plate at a density of 1 × 10^5^ cells per well and treated with LPS (InvivoGen, 6 μg/mL) with or without 5% Ly-H-PRF for 24 h. Following treatment, cells were collected and washed three times with PBS. Subsequently, cells were stained with PE anti-mouse CD206 (clone C068C2, 1:100, BioLegend) and FITC anti-mouse CD86 (clone C105005, 1:100, BioLegend) antibodies, and then incubated at 4 °C for 30 min. After incubation, cells were washed three times with PBS, resuspended in PBS, and subjected to flow cytometric analysis using a BD Fortessa™ X-20 instrument. FlowJo 10.8.1 software was used for data analysis.

### 2.7 Transwell assay

L929 cells were starved for 12 h, collected, and seeded into the upper chambers of transwell plates (Corning, 24-well plate, 8 μm pore size) at a density of 5 × 10^4^ cells per well. LPS (InvivoGen, 6 μg/mL) with or without 5% Ly-H-PRF was added to the wells of the 24-well transwell plates. After 12 h, the upper chamber was taken out, and the cells were fixed with 4% formaldehyde. Cotton swabs were used to remove non-migrated cells from the upper surface of the upper chamber. The cells were then stained with 0.5% crystal violet (Sigma, United States) for 30 min, washed three times with PBS, and the migrated cells on the lower surface of the upper chamber were recorded and counted under an optical microscope (Nikon NI-SS, Japan) for statistical analysis.

### 2.8 Scratch wound healing assay

L929 cells were seeded in 6-well plates at a density of 3 × 10^5^ cells per well. After 24 h, a scratch was made using a sterile 3 mm diameter pipette tip. Images were captured under an optical microscope (Nikon DS-Qi2, Japan) immediately after scratching (0 h). The cells were then treated with LPS (InvivoGen, 6 μg/mL) with or without 5% Ly-H-PRF for 12 h, and images were captured again. The migration rate was calculated as follows:

$$\frac{\left(Scratch\;width\;at\;0\;hour\right)-\left(Scratch\;width\;at\;12\;hours\right)}{Scratch\;width\;at\;0\;hours}\times 100\%$$

### 2.9 *In vivo* infected burn wound model

The experimental protocol was approved by the Ethics Committee of Wuhan University, ensuring that the experiment complies with ethical standards. Infected burn wound model was created on the dorsal spine of mice (C57BL/6). Mice were anesthetized via intraperitoneal injection of pentobarbital solution (4%)and then depilated and disinfected. Two scald wounds were made on each side of the dorsal spine using a circular probe with a diameter of 1.5 cm, heated in hot water to 100 °C, and applied to the depilated and disinfected skin tissue for 10 s. After 24 h, the scabs were removed. S.a bacterial liquid was prepared in advance and diluted to 1 × 10^7 colony-forming units (cfu)/ml. Mice were randomly divided into four groups: PBS group (Burn wound with PBS), S.a group (Burn wound with S.a), S.a+BO group (Burn wound with S.a and Burn Ointment), and S.a+ Ly-H-PRF group (Burn wound with S.a and Ly-H-PRF), with six wounds in each group.

In the PBS group, the burn wounds were covered with sterile gauze soaked in physiological saline and then wrapped with bandages. In the S.a group, sterile gauze cut to the size of the wound was placed on the wound, and then 10 μL of prepared bacterial liquid was dropped onto the gauze before wrapping with bandages. In the S.a+BO group, after applying the bacterial liquid, burn ointment (Jingwanhong, Tianjin, China) was applied to the gauze before wrapping with bandages. In the S.a+ Ly-H-PRF group, after applying the bacterial liquid, 10 μg of PRF lyophilized powder was added to the gauze before wrapping with bandages. After wound formation, the wounds were cleaned with sterile physiological saline every 2 days, disinfected with povidone-iodine, and then new dressings were applied. The healing rate was calculated as follows: Healing rate = ((wound area on day 1) - (wound area on day 5))/(wound area on day 1) × 100%.

### 2.10 Histological analysis

On the fifth day, full-thickness tissue samples of the wound were collected from each mouse (n = 6 mice per group), fixed in neutral buffered formalin for 48 h, and embedded in paraffin. One representative wound tissue block per mouse were sectioned to prepare 5 μm sections. Hematoxylin and eosin (HE) staining was performed to observe the re-epithelization and wound healing (one analysis per mouse). Masson’s trichrome staining was performed to observe the deposition of collagen fibers at the wound site (one analysis per mouse). Immunofluorescence staining was conducted to compare the polarization of macrophages at the wound site among different groups. For immunofluorescence staining, the slides were blocked with 1% bovine serum albumin. PE anti-mouse CD206 (clone C068C2, 1:100, BioLegend) and FITC anti-mouse CD86 (clone C105005, 1:100, BioLegend) were applied at 4 °C overnight. Images were captured using a fluorescent microscope (Zeiss LSM880 with Airyscan, Germany). Multiple fields of view were analyzed per immunofluorescence section to assess macrophage polarization within the single wound sample from each mouse.

### 2.11 Statistical analysis

All the experiments were analyzed using a two-way Student’s t-test. For the comparison of different specimens, the unpaired t-test was used. For the comparison of different treatments within the same specimen, the paired t-test was used. P values of less than 0.05 were considered statistically significant. In the figures, asterisks indicate *P < 0.05, **P < 0.01, and ***P < 0.001, ****P < 0.0001, and ns = not significant.

## 3 Results

### 3.1 Photos, microscopic morphology, and solubility of the materials

**FIGURE 1:**
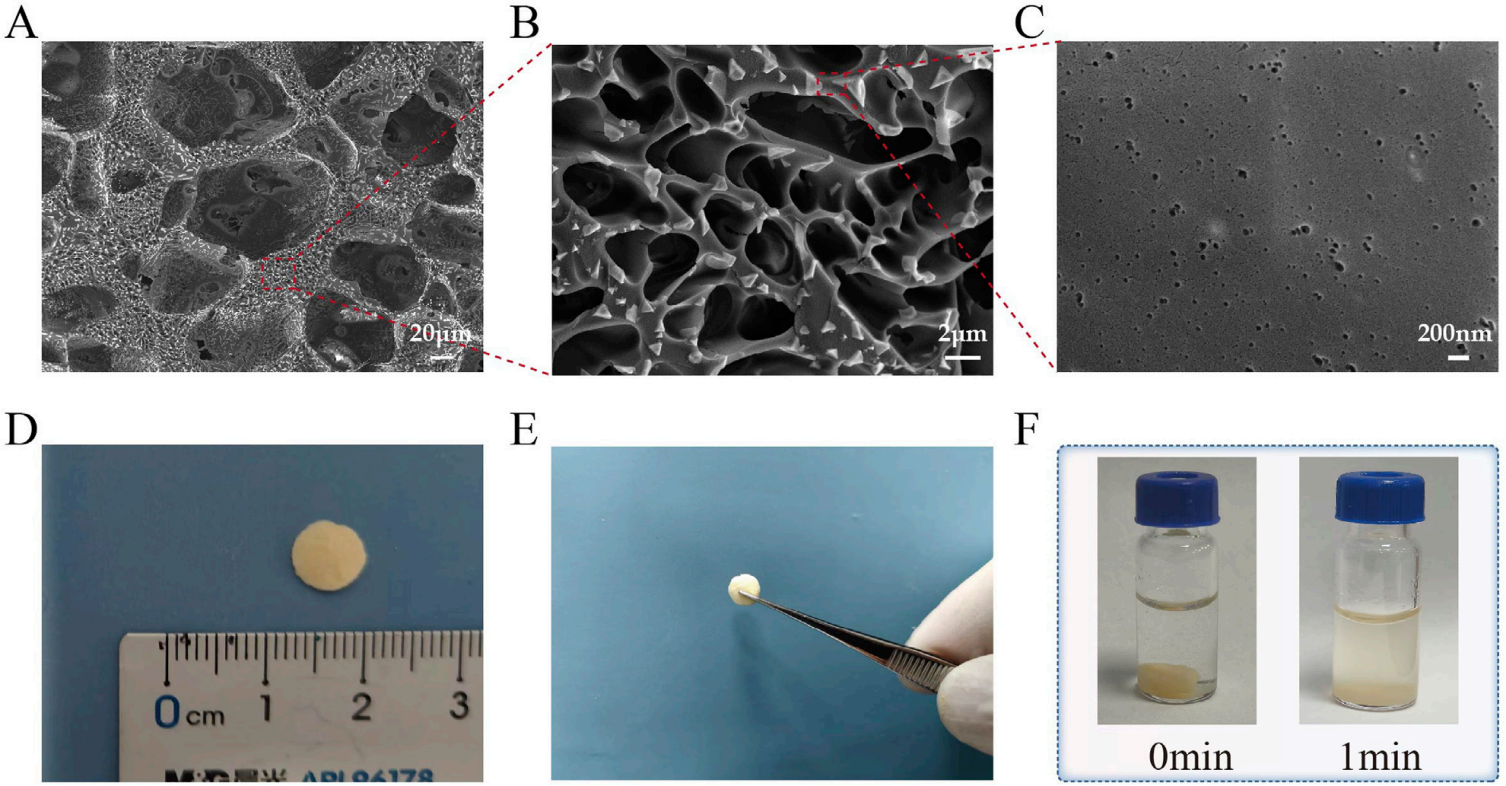
Photos, microscopic morphology, and solubility of the materials. **(A–C)** The scanning electron microscope (SEM) image of Ly-H-PRF at magnifications of ×300 , 5,000 × , and 30,000; **(D)** The typical sizes of materials used in applications; **(E)** Ly-H-PRF can be fully handled or picked up; **(F)** The solubility of the material in PBS (phosphate-buffered saline).

As shown in Figure 1, after lyophilization, the material forms sheet-like structures that can be easily handled and used. The material exhibits a sponge-like porous structure at ×30,000 magnification in SEM observation. It can be easily handled as a whole and readily dissolves in PBS.

### 3.2 Antibacterial effect of ly-H-PRF

**FIGURE 2:**
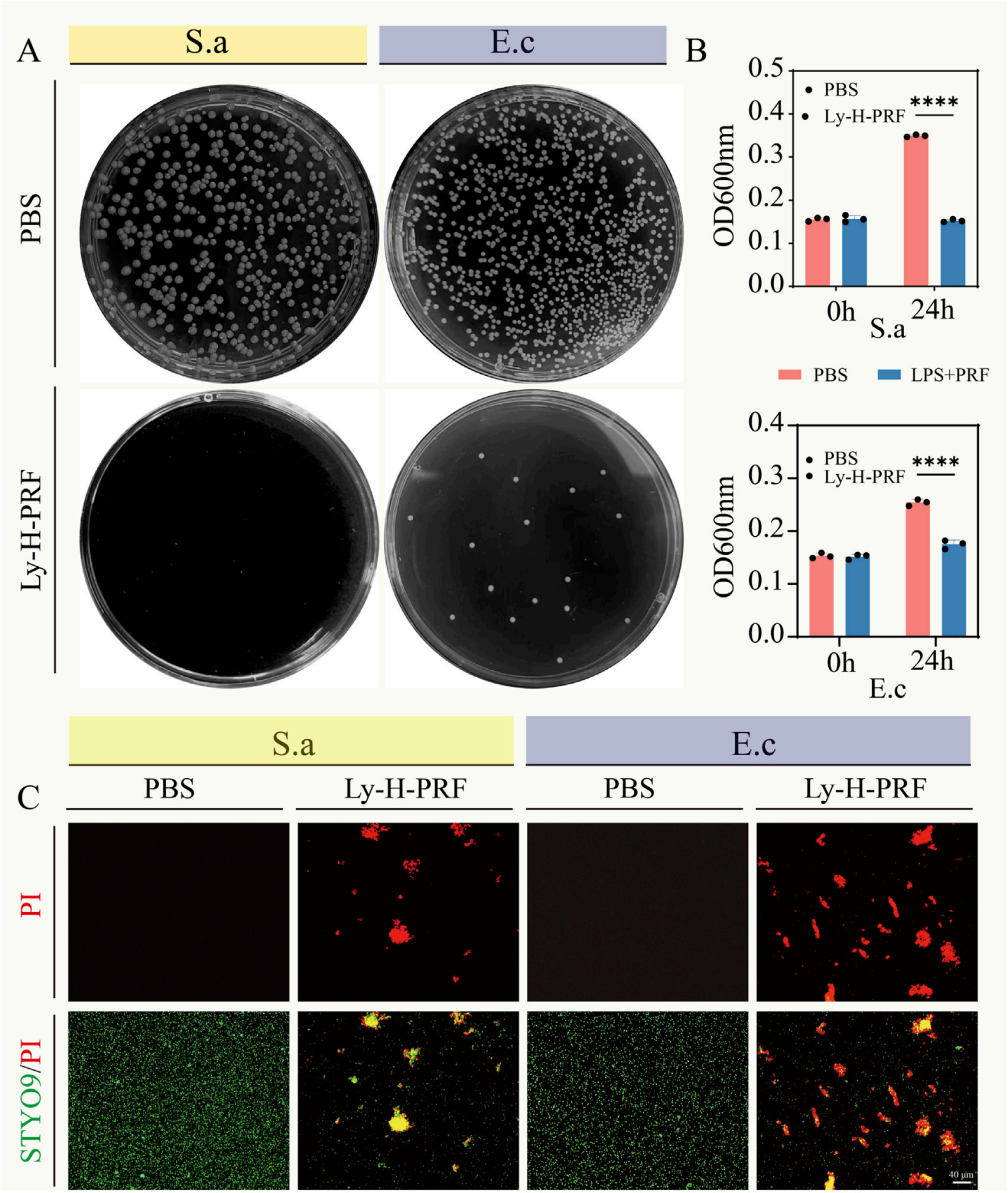
*In vitro* antibacterial activity of Ly-H-PRF. **(A)** Representative optical images of colony-forming units for *Staphylococcus aureus* suspensions and *Escherichia coli* suspensions treated with or without Ly-H-PRF. **(B)** Quantitative analysis of OD600 of *Staphylococcus aureus* and *Escherichia coli* treated with or without Ly-H-PRF; **(C)** Fluorescence images show the results of the live/dead staining, with viable bacteria appearing green and dead bacteria appearing red.

As shown in Figure 2A, after 24 h of incubation with a 10% Ly-H-PRF mixture in the bacterial suspension, the number of bacterial colonies on the agar plates was significantly lower compared to the control group, for both E.cc and S.a. Additionally, the absorbance values of the bacterial suspensions measured after 24 h of incubation showed the same trend, with the suspensions treated with 10% Ly-H-PRF appearing noticeably clearer, indicating reduced bacterial growth, a difference that was statistically significant (Figure 2B). The results of the live/dead bacterial staining further confirmed this, showing a large number of dead bacteria in the 10% Ly-H-PRF-treated group, with fewer live bacteria. The dead bacteria appeared to aggregate and form clusters (Figure 2C).

### 3.3 The effect of ly-H-PRF on LPS-induced apoptosis of macrophages and macrophages cell cycle

**FIGURE 3:**
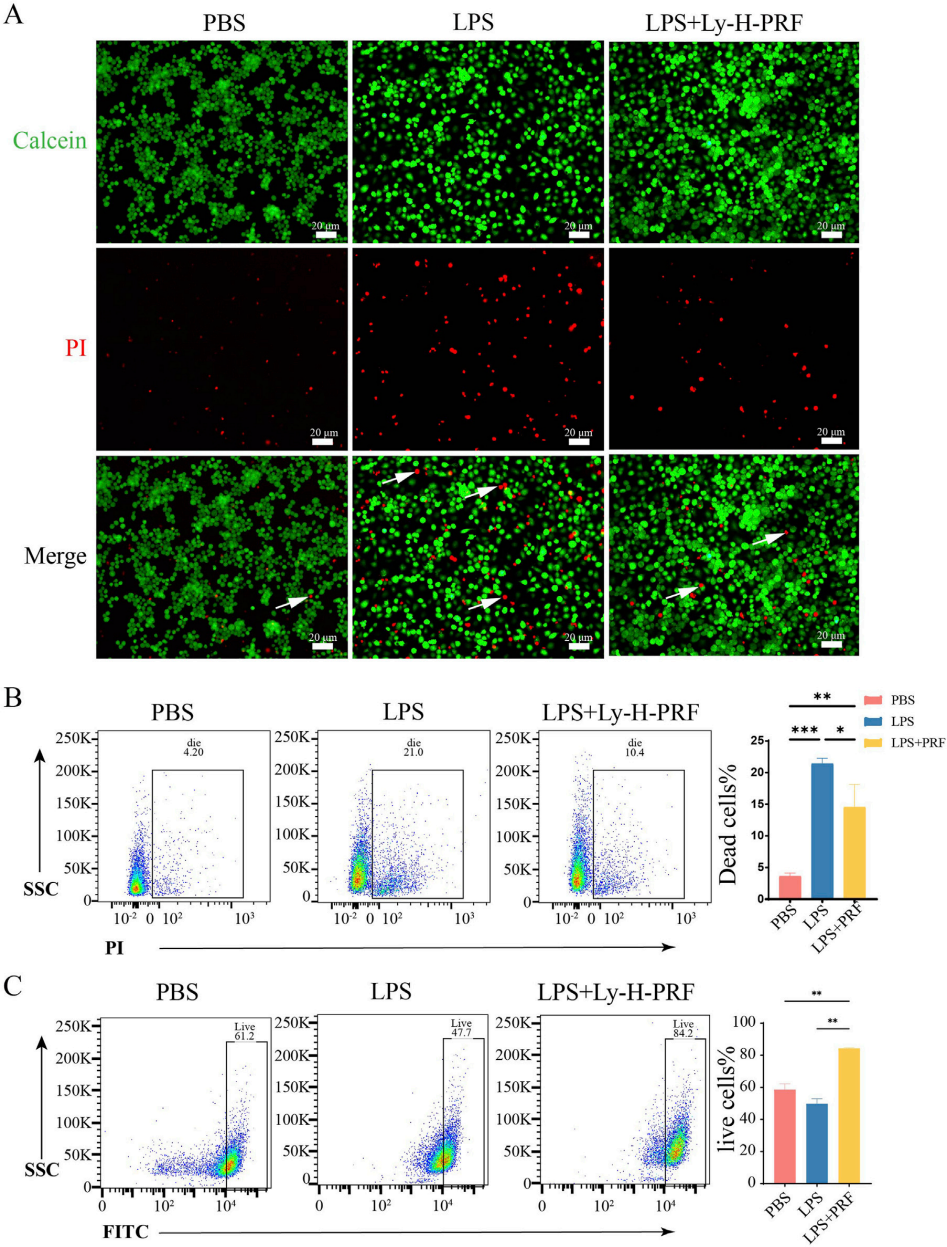
Live/dead assay of RAWs treated with/without LPS and Ly-H-PRF at 24 h. **(A)** Fluorescence images show the results of the live/dead staining with viable cells appear green and dead cells (white arrow) appear red. Scale bars = 20 μm; **(B)** Representative images and quantitative analysis of dead cells measured by flow cytometry; **(C)** Representative images and quantitative analysis of live cells measured by flow cytometry.

From the fluorescent and flow cytometry images in Figure 3, it was observed that the number of dead macrophages was significantly higher in the LPS group when compared to the control group. However, the addition of Ly-H-PRF was found to significantly decrease the number of these dead cells caused by LPS, suggesting that Ly-H-PRF could rescue macrophage apoptosis induced by bacterial endotoxins. Additionally, Ly-H-PRF stimulation also significantly increased the number of viable macrophages, possibly due to the growth factors present in Ly-H-PRF found to stimulate cell proliferation.

### 3.4 Cell cycle and apoptosis of RAWs treated with/without LPS and Ly-H-PRF at 24 h

**FIGURE 4:**
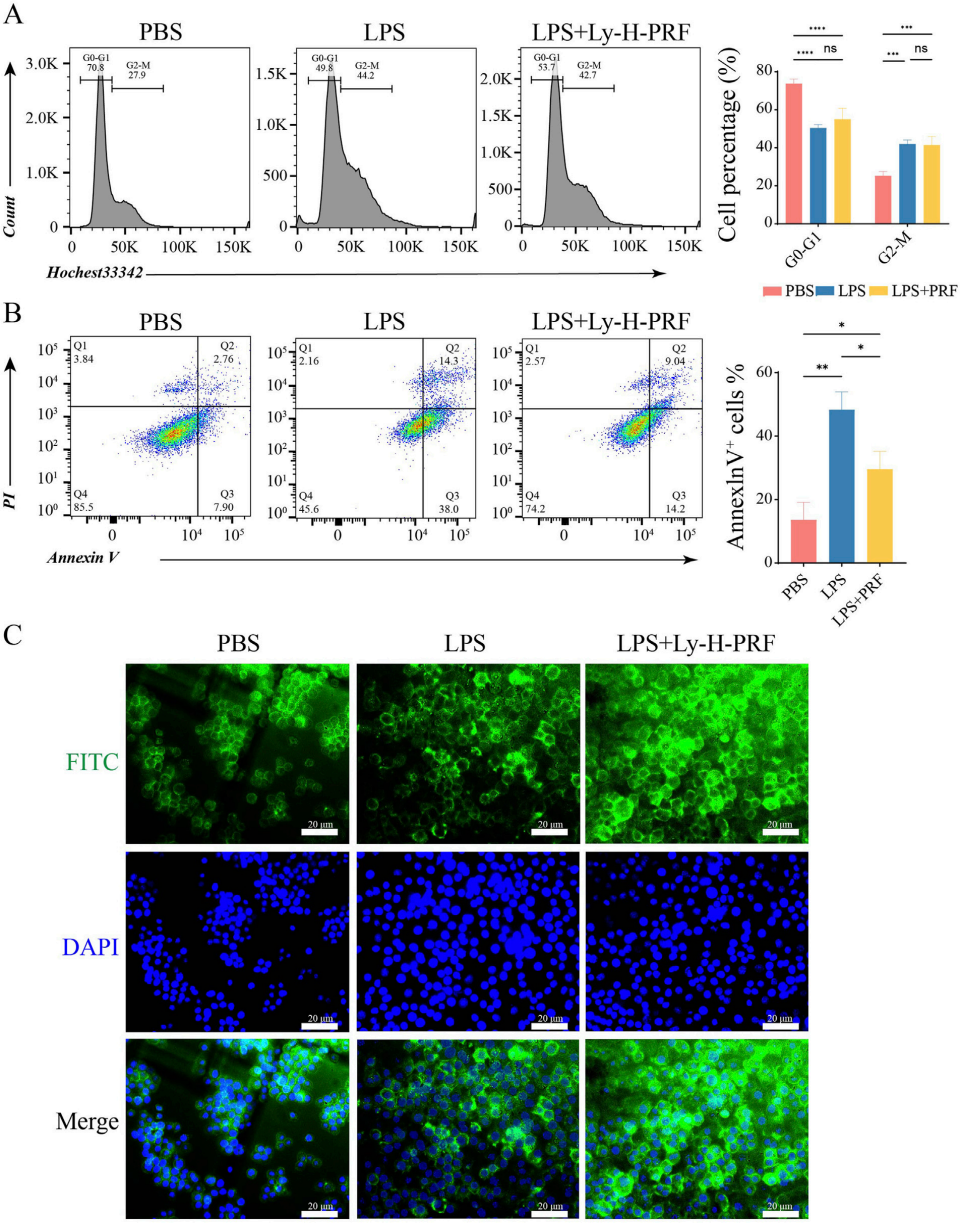
Cell cycle and apoptosis of RAWs treated with/without LPS and Ly-H-PRF at 24 h **(A)** Diagram and statistical analysis of cell cycle analysis in RAW 264.7 cells by flow cytometry. **(B)** Representative images and statistical analysis of apoptosis analysis in RAW 264.7 cells by flow cytometry. **(C)** Representative images of cytoskeleton in RAW 264.7 cells. Scale bars = 20 μm.

In Figure 4A, it was observed that compared with the control group, both the LPS group and the LPS + Ly-H-PRF group promoted the transition of macrophages from the G0-G1 phase to the G2-M phase. Figure 4B demonstrates that the LPS group exhibited a significant increase in apoptosis compared to the control group, while the LPS + Ly-H-PRF group showed a significant decrease in apoptosis compared to the LPS group. This further confirms the protective effect of Ly-H-PRF on LPS-induced macrophage apoptosis. Cellular morphology staining showed no differences in the cytoskeletal morphology of the three groups of cells at 24 h (Figure 4C).

### 3.5 The effect of ly-H-PRF on macrophage polarization

**FIGURE 5:**
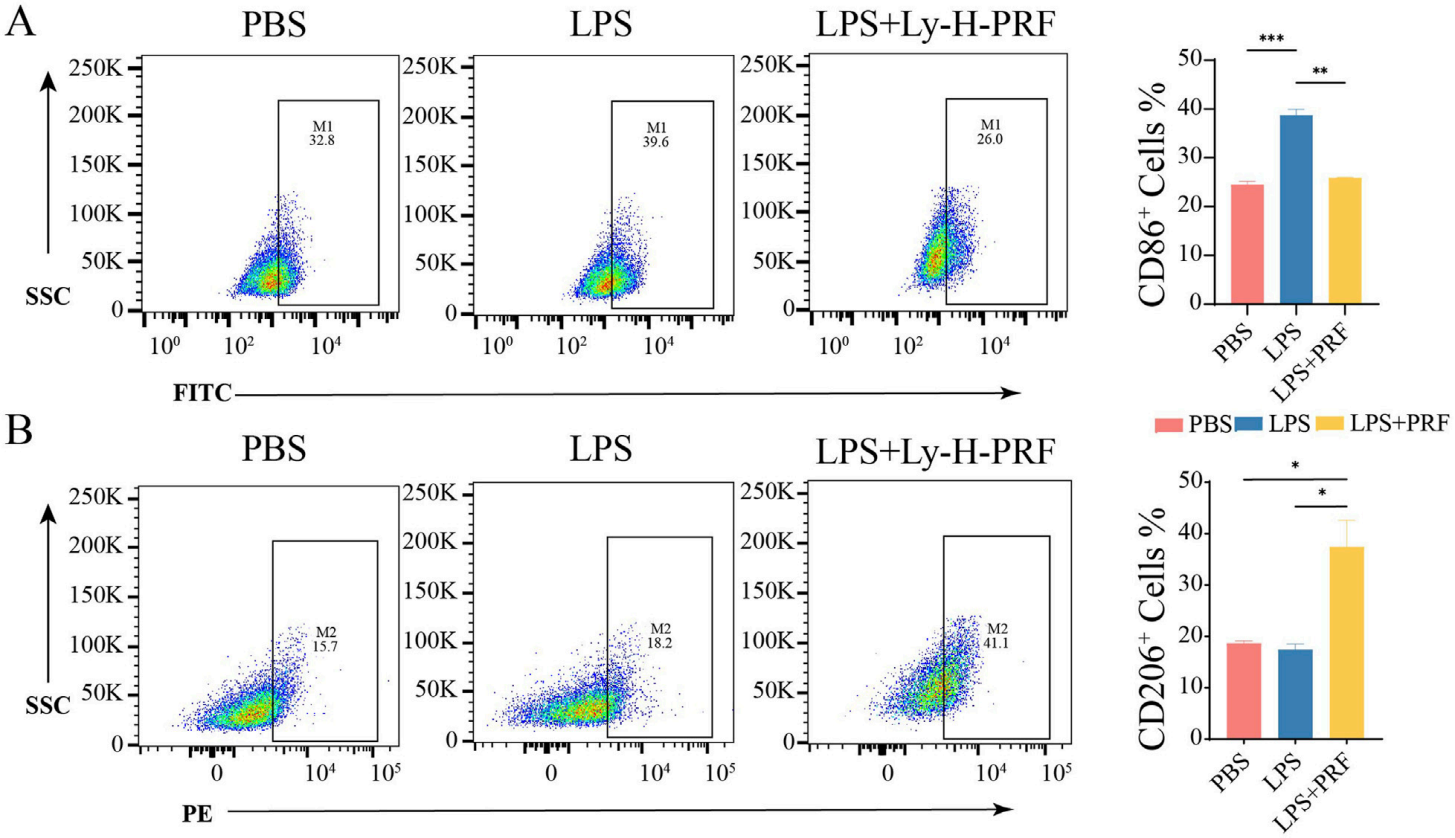
Effect of PRF on polarization of RAW treated with LPS and Ly-H-PRF at 24 h. **(A)** Representative images and ratio of CD86^+^ cells measured by flow cytometry. **(B)** Representative images and ratio of CD206+ cells measured by flow cytometry.

The effect of Ly-H-PRF on macrophage polarization was assessed using flow cytometry to detect macrophage surface polarization markers. It was found that after LPS stimulation, the M1 marker CD86 on macrophages was significantly increased, while the addition of Ly-H-PRF significantly decreased its level, restoring it to the levels similar to those observed in the control group (Figure 5A). Flow cytometry results also showed that LPS treatment did not affect the expression of the M2 marker CD206 on macrophages, but Ly-H-PRF treatment significantly increased the expression of this marker compared to LPS and control groups (Figure 5B). This suggests that PRF treatment may assist in the formation of an immune microenvironment that promotes wound healing by promoting M2 macrophage polarization.

### 3.6 The effect of ly-H-PRF on LPS-induced apoptosis and migration of fibroblasts

**FIGURE 6:**
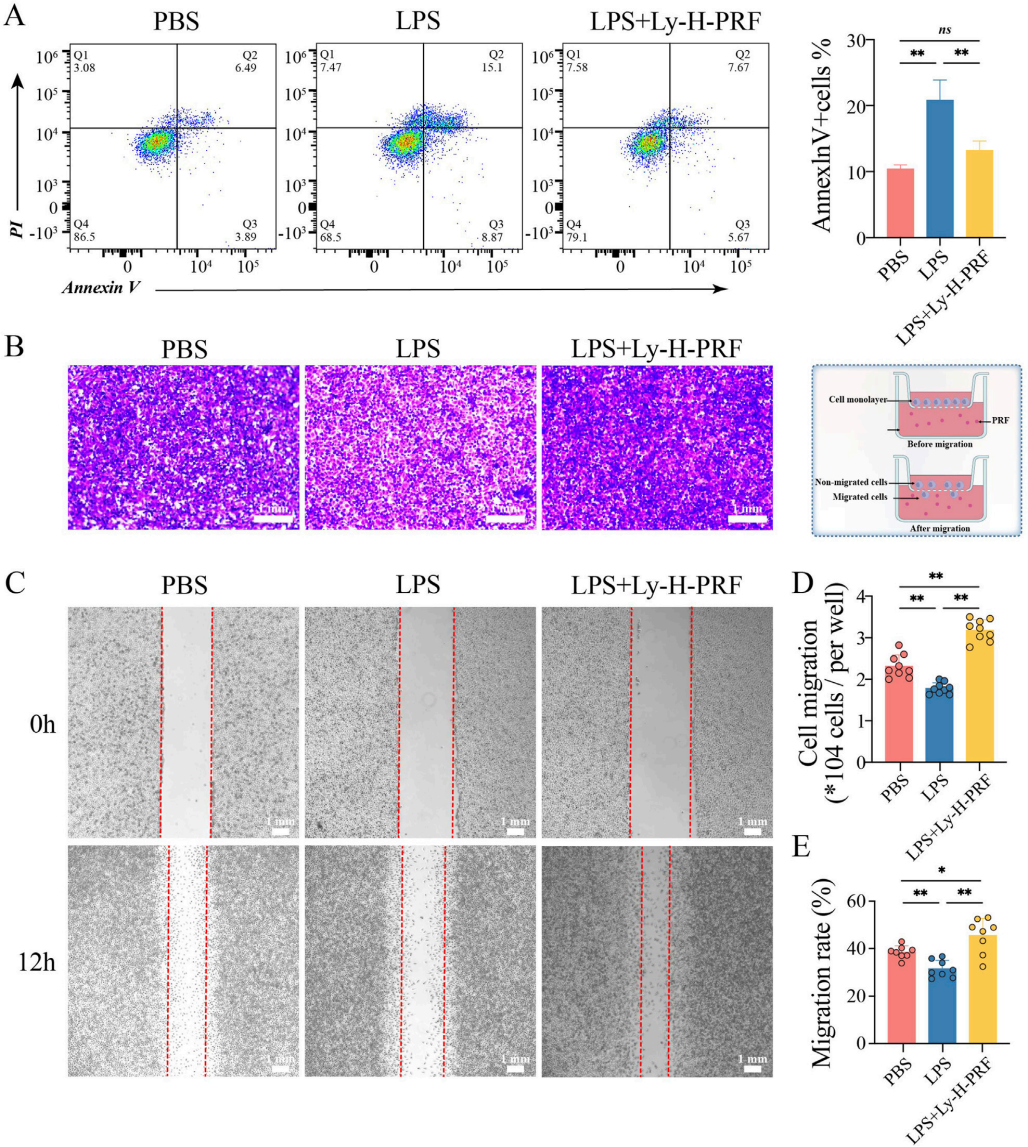
Apoptosis and Migration of L929 treated with/without LPS and Ly-H-PRF at 24 h. **(A)** Representative images and quantitative analysis of apoptosis analysis in L929 cells by flow cytometry. **(B,C)** Representative images and quantitative analysis of transwell assay in L929 cells **(D,E)** Representative images and quantitative analysis of scratch assay in L929 cells.

Flow cytometry was further used to assess apoptosis of L929 cells after 24 h of LPS stimulation. It was found that apoptosis rate of fibroblasts significantly increased and doubled after LPS treatment compared to the control group, while treatment with Ly-H-PRF significantly decreased this rate, restoring it to nearly pre-treatment levels (Figure 6A). Transwell experiments demonstrated that after LPS treatment, the migration ability of L929 cells significantly decreased, whereas Ly-H-PRF treatment not only significantly rescued their migration ability but also significantly increased it to levels higher than those observed prior to treatment (Figures 6B,C). Results from scratch assays were similar to those from Transwell experiments (Figures 6D,E), indicating that LPS inhibited the migration ability of fibroblasts, while Ly-H-PRF restored the impaired migration ability caused by LPS and even further enhanced it.

### 3.7 The effect of ly-H-PRF on the healing of infected burn wounds

**FIGURE 7:**
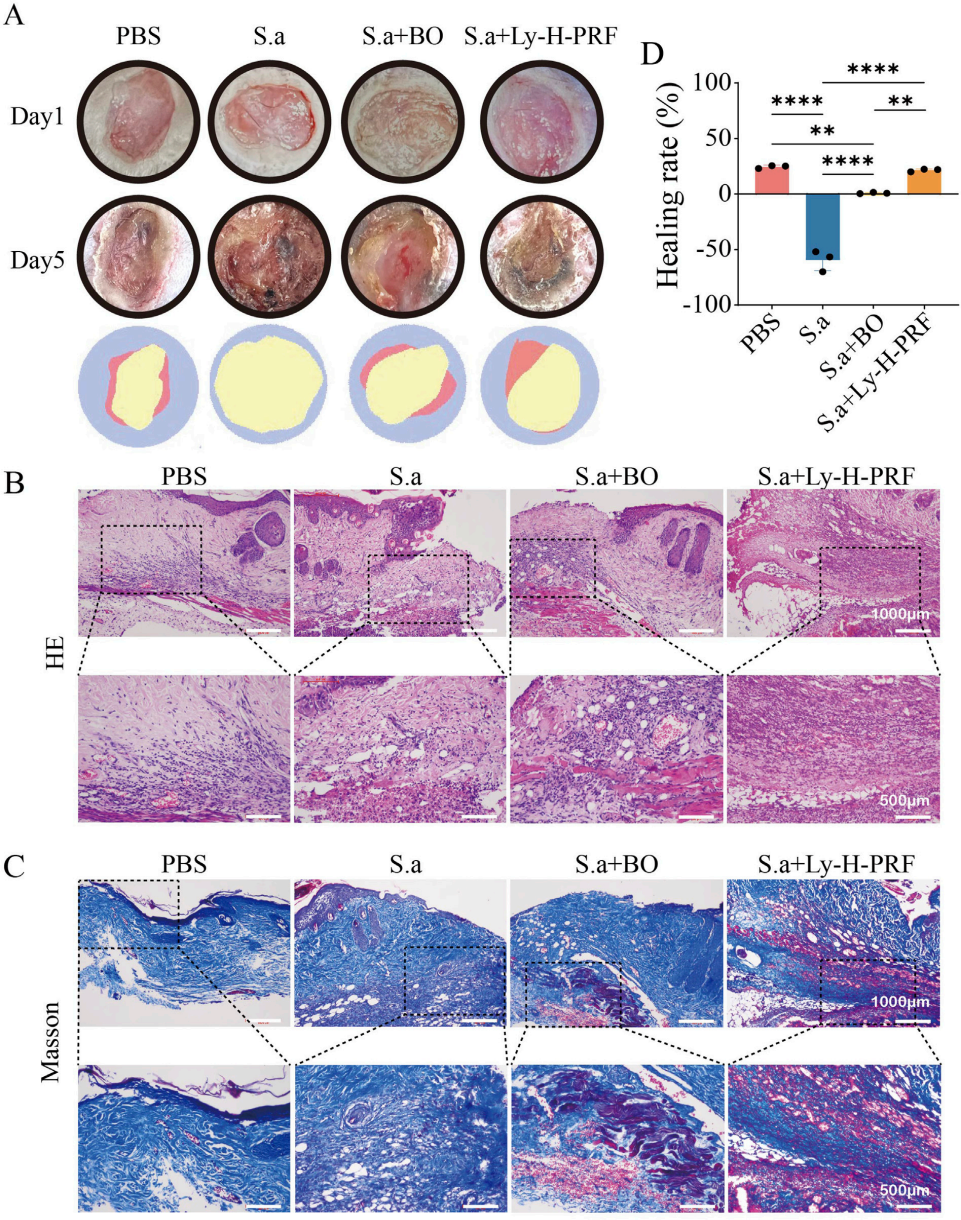
Infected burn wound healing in different groups on day 0 and day 5. **(A)** Representative photographic images of the burn wounds in different groups on day 0 and 5, respectively. The error bars represent SD (n = 6), and the statistics are determined by t-test (*P i 0.05). **(B)** Quantification of the wound healing in each group on day 0 and 5. **(C)** H&E staining of wounds at day 5. High magnified view of the image shown in lower row. The scale bar are 1,000 μm (upper row) or 500 μm (lower row). **(D)** Masson’s trichrome staining of wounds at day 5. High magnified view of the image shown in lower row.

In the mouse infected, burn wound model, it was observed that without any treatment after burn wound creation, there was a slight decrease in wound area and a small amount of scab formation after 5 days. However, in the S.a group, the wound area significantly increased due to the infection. In the group treated with commercially available burn ointment (S.a+BO group) during infection, there was no significant change in wound area, and some necrotic tissue was observed. Conversely, simultaneous treatment with Ly-H-PRF during infection resulted in significant scab formation, and the reduction in wound area was similar to that of the uninfected group, further demonstrating that a protective effect of Ly-H-PRF was observed on infected burn wounds (Figures 7A,B). HE staining and Masson’s trichrome staining both showed abundant vacuolar structures in the burn tissue of the S.a group, possibly resulting from tissue damage caused by bacteria. In the S.a+BO group, there were fewer vacuolar structures, and some newly formed collagen tissue was observed. In the S.a+ Ly-H-PRF group, there were abundant newly formed collagen fibers, with fewer observed vacuolar structures. Moderate infiltration of inflammatory cells was observed in the PBS group, while severe infiltration of inflammatory cells was observed in the S.a group. However, inflammation infiltration decreased after treatment with burn ointment and especially in the Ly-H-PRF group (Figures 7C,D).

### 3.8 Effect of Ly-H-PRF on macrophage polarization in infected burn wound models

**FIGURE 8:**
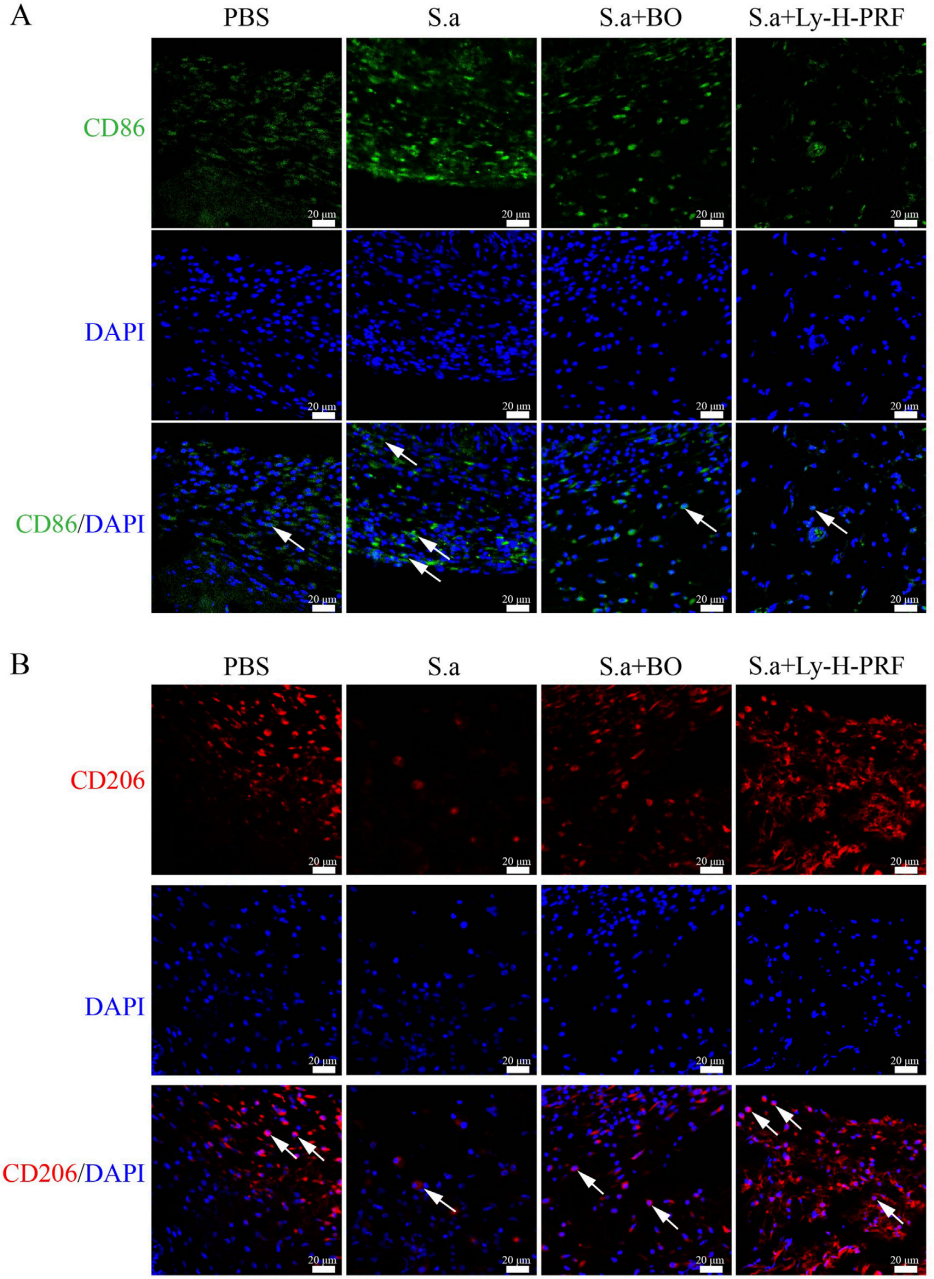
Effect of Ly-H-PRF on macrophage polarization in infected burn wound models. **(A)** Immunofluorescence staining of M1 macrophage-related marker CD86 in the wound tissue on day 5. Scale bars = 20 μm. **(B)** Immunofluorescence staining of M2 macrophage-related marker CD206 in the wound tissue on day 5. Scale bars = 20 μm.

In the mouse infected burn wound model, it was observed that after 5 days of infection in the S.a group, there was a increase in CD86-positive cells in the burn tissue, indicating that infection led to polarization of macrophages towards the M1 phenotype. The number of M1 macrophages in the S.a+BO group was lower than that in the S.a group, and further decreased in the S.a+ Ly-H-PRF group (Figure 8A). On the other hand, M2 macrophages were decreased in the burn tissue of the S.a group, while the number of M2 macrophages in the S.a+BO group was increased compared to the S.a group, and further increased in the S.a+Ly-H-PRF group. This suggests that the use of Ly-H-PRF was able to reduce excessive inflammatory responses and promote soft tissue healing (Figure 8B).

### 3.9 Ly-H-PRF promotes infected burn wound epithelialization and vascular regeneration

**FIGURE 9:**
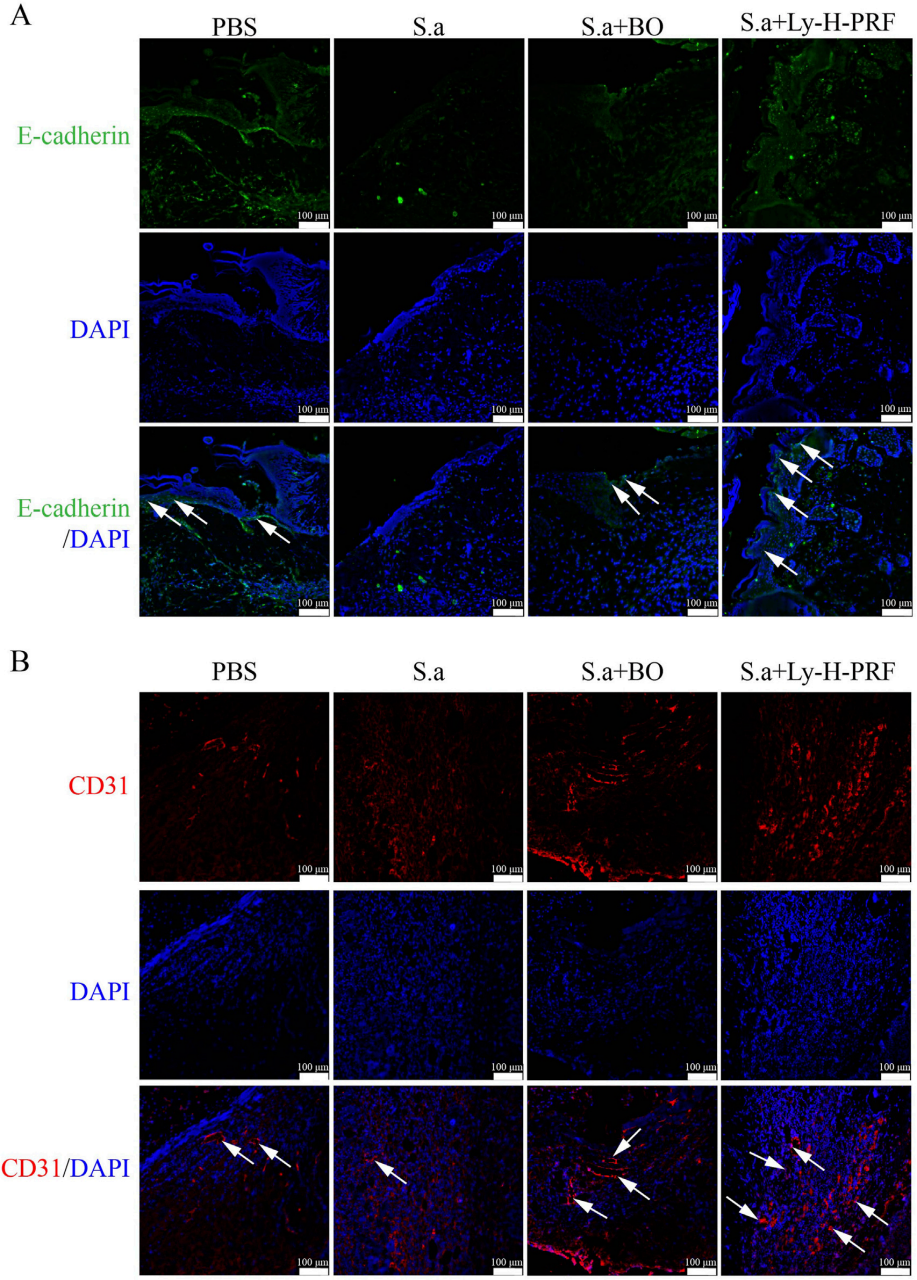
Ly-H-PRF promotes infected burn wound epithelialization and vascular regeneration. **(A)** Immunofluorescence staining of epithelialization marker E-cadherin in the wound tissue on day 5. Scale bars = 100 μm. **(B)** Immunofluorescence staining of CD31 in the wound tissue on day 5. Scale bars = 100 μm.

In the mouse infected burn wound model, we observed that after 5 days of infection in S.a group, the expression of E-cadherin, an epithelialization marker in the wound tissue of S.a+BO group, was higher than that of S.a group, while the expression of E-cadherin in S.a+Ly-H-PRF group was significantly higher than that of S.a group. This suggests that the use of Ly-H-PRF promotes epithelialization of infected burn wounds (Figure 9A). On the other hand, the expression of CD31 in wound tissue of S.a+BO group was higher than that of S.a group, and the expression of CD31 in S.a+Ly-H-PRF group was significantly higher than that of S.a group, indicating that Ly-H-PRF could promote vascular regeneration in infected burn wounds (Figure 9B).

## 4 Discussion

When a scald occurs, the skin barrier is compromised, making it susceptible to the entry of exogenous pathogens, leading to challenging-to-treat infections. Studies have shown that approximately 42%–65% of burn-related deaths are associated with infection (Lachiewicz et al., 2017). Therefore, developing solutions for treating infected burn wounds is highly important.

Platelet concentrates are biological materials rich in growth factors and viable cells, and they feature a three-dimensional fibrin scaffold. Due to the variety of growth factors present, they find extensive applications in skin regeneration, dental regeneration, sports medicine, plastic surgery, and various burn cases, exhibiting favorable outcomes (Lachiewicz et al., 2017; Tavousi et al., 2024).The application of novel H-PRF in infected burn wounds has not yet been reported. In this study, we utilized H-PRF and preserved it by freeze-drying, offering the advantage of convenient usage. Furthermore, it can be sampled multiple times for preparation, thus circumventing the issue of insufficient autograft sources.

Macrophages play crucial roles in wound healing, host immunity, and immune regulation. They can polarize into pro-inflammatory M1-type and pro-tissue regeneration M2-type macrophages, regulating different tissue healing processes (Xiong et al., 2023; Kharaziha et al., 2021). During the burn wound healing phase, if bacterial infection occurs in patients, it inhibits the conversion of M1 to M2 macrophages, thereby impeding the transition of the wound into tissue remodeling and regeneration phases. Inducing the transformation of M1 cells into M2 cells in the wound presents a challenge for burn healing (Wu et al., 2022; Schwacha, 2003; Huang et al., 2023). In this study, LPS was used to stimulate RAW 264.7 macrophage cells *in vitro*, simulating an inflammatory environment *in vivo* (Shi et al., 2022). LPS stimulation was found to induce apoptosis in macrophages and promote M1 polarization, while the use of LY-H-PRF was shown to reverse this phenotype and increase the quantity of M2 cells. The mouse-infected burn wound model also validated these findings. In a S.aureus-infected burn model, treatment with Ly-H-PRF reduced the presence of M1 macrophages and increased M2 macrophages in these infected burn wounds. This suggests that Ly-H-PRF was able to improve the sustained inflammation in these burn wounds with simultaneous bacterial infection, leading to difficult-to-heal conditions. Corroborating our results, Nasirzadeh et al. also showed that H-PRF can modulate the inflammatory response by shifting macrophage polarization from the pro-inflammatory M1 phenotype to the anti-inflammatory M2 phenotype (Nasirzade et al., 2020). Fibroblast proliferation and migration also play significant roles in wound healing (Oryan et al., 2019) and it was found that LPS stimulation induces apoptosis in L929 cells in this study, whereas Ly-H-PRF powder could also prevent cell death under these conditions. Furthermore, through transwell and scratch assays, we discovered that Ly-H-PRF could also promote fibroblast migration, further indicating its potential to facilitate tissue healing in infected wounds.

In this experiment, we utilized a burn model infected with S.a, a widely employed model for studying post-burn infections and treatment efficacy. As previous studies have demonstrated, without proper treatment, infected burn wounds may continue to deteriorate and increase in size due to immune system dysregulation (Shahriari-Khalaji et al., 2023). This immune dysregulation manifests as dysfunction of local immune cells and exacerbation of inflammatory responses, hindering the wound healing process. We used a commercially available burn ointment containing a combination of traditional Chinese medicine ingredients as the positive control, which effectively alleviates post-burn infections and accelerates wound healing. However, we found that treatment with Ly-H-PRF powder yielded more significant results. This may be attributed to the potent antimicrobial properties of H-PRF (Feng et al., 2020; Moraschini et al., 2000), which also significantly promotes wound healing.

Lastly, it remains crucially important to point out that the centrifugation protocols, tube types utilized, and medical device utilized for the production of PRF all matter significantly in order to maximize the regenerative potential of PRF. In a recent study titled “Optimization of Platelet Rich Fibrin”, a series of key features were discussed to elevate clinical use of PRF in private practice (Miron et al., 2000). One important feature that has been discussed was the effect of PRF tubes on the final outcomes of PRF (Miron et al., 2000; Miron et al., 2022; Miron et al., 2021; Wei et al., 2024). Many PRF tubes, though marketed as potentially being “chemical-free”, are loaded with chemical additives such as silica or silicone, which may negatively impact the final production of PRF (Miron et al., 2021; Masuki et al., 2020; Tsujino et al., 2019a; Tsujino et al., 2019b).

## 5 Conclusion

The present study demonstrated that Ly-H-PRF is able to exert antibacterial effects, modulate macrophage polarization from the M1 to M2 phenotype, and enhance skin fibroblast migration. It also showed promising efficacy in treating infected burn wounds by promoting wound healing, reducing inflammatory cell infiltration, and increasing collagen synthesis. These findings provide proof-of-concept for the clinical use of Ly-H-PRF as a cost-effective and convenient treatment for infection control and tissue regeneration in infected burn wounds. However, its safety, efficacy, and long-term outcomes require further investigation. This study also supports further exploration of Ly-H-PRF in other regenerative medicine applications.

## Data Availability

The raw data supporting the conclusions of this article will be made available by the authors, without undue reservation.
